# Hydrogel viscoelasticity modulates migration and fusion of mesenchymal stem cell spheroids

**DOI:** 10.1002/btm2.10464

**Published:** 2022-12-27

**Authors:** David T. Wu, Mani Diba, Stephanie Yang, Benjamin R. Freedman, Alberto Elosegui‐Artola, David J. Mooney

**Affiliations:** ^1^ Laboratory for Cell and Tissue Engineering, John A. Paulson School of Engineering and Applied Sciences Harvard University Cambridge Massachusetts USA; ^2^ Wyss Institute for Biologically Inspired Engineering, Harvard University Boston Massachusetts USA; ^3^ Department of Oral Medicine, Infection, and Immunity Harvard School of Dental Medicine Boston Massachusetts USA; ^4^ Department of Dentistry‐Regenerative Biomaterials Radboud Institute for Molecular Life Sciences, Radboud University Medical Center Nijmegen the Netherlands; ^5^ Present address: Cell and Tissue Mechanobiology Laboratory The Francis Crick Institute London UK; ^6^ Present address: Department of Physics King's College London London UK

**Keywords:** biomaterials, cell migration, extracellular matrix, hydrogels, mechanotransduction, stem cell spheroids, viscoelasticity

## Abstract

Multicellular spheroids made of stem cells can act as building blocks that fuse to capture complex aspects of native in vivo environments, but the effect of hydrogel viscoelasticity on cell migration from spheroids and their fusion remains largely unknown. Here, we investigated the effect of viscoelasticity on migration and fusion behavior of mesenchymal stem cell (MSC) spheroids using hydrogels with a similar elasticity but different stress relaxation profiles. Fast relaxing (FR) matrices were found to be significantly more permissive to cell migration and consequent fusion of MSC spheroids. Mechanistically, inhibition of ROCK and Rac1 pathways prevented cell migration. Moreover, the combination of biophysical and biochemical cues provided by fast relaxing hydrogels and platelet‐derived growth factor (PDGF) supplementation, respectively, resulted in a synergistic enhancement of migration and fusion. Overall, these findings emphasize the important role of matrix viscoelasticity in tissue engineering and regenerative medicine strategies based on spheroids.

## 1 INTRODUCTION

Cell migration is a critical process in wound healing and regeneration of various tissues.^1^ In living tissues, cells are physically confined within a complex and three‐dimensional (3D) extracellular matrix (ECM) to which they are mechanically coupled by adhesion molecules such as integrins. These molecules enables communication and transmission of forces between the cells and the ECM.^1^, ^2^ Accordingly, numerous previous studies have focused on the effect of matrix elasticity or stiffness on cell behavior.^1^, ^3^, ^4^, ^5^, ^6^, ^7^ Nevertheless, the dynamic character of the ECM, even in hard tissues, endows it with an ability to respond to mechanical deformation/force in a time‐dependent manner (i.e., viscoelasticity).^8^, ^9^, ^10^, ^11^, ^12^, ^13^, ^14^, ^15^, ^16^, ^17^ Recent studies have revealed that matrix viscoelasticity regulates multiple cellular processes, including spreading, differentiation, and migration.^8^, ^9^, ^10^, ^11^, ^12^, ^14^ In particular, these findings have shed light on the impact of the viscous character of these matrices by highlighting the role of stress relaxation (i.e., time‐dependent decrease in stress under a constant deformation of the matrix). For instance, when embedded within hydrogels with a similar elasticity, fibroblasts exhibited enhanced spreading in the hydrogels with a more rapid stress relaxation behavior.^12^

Multicellular spheroids and organoids have been shown to be effective tools for in vitro modeling and in vivo regeneration of tissue defects.^17^, ^18^, ^19^ In particular, spheroids made of mesenchymal stem cells (MSC) have demonstrated promising results for in vitro biofabrication of tissue‐like constructs or in vivo regeneration of tissue defects.^7^, ^18^, ^20^, ^21^, ^22^ These cellular clusters are often embedded in hydrogel matrices to enable inter‐spheroid fusion, thereby capturing complex aspects of native in vivo environments such as spatial organization and cellular heterogeneity.^19^, ^23^, ^24^, ^25^, ^26^ For the biofabrication of in vitro tissue models, recent investigations have successfully demonstrated formation of tissue‐like constructs by spatial positioning of multiple spheroids within a hydrogel matrix.^24^, ^25^, ^26^ In these strategies, the ability of neighboring spheroids to interact and fuse together plays a pivotal role in engineering the formation of complex tissue models. These fusion phenomena largely depend on inter‐spheroid cell migration resulting in the formation of cellular bridges between neighboring spheroids.^27^ Similarly, based on the fusion of multiple organoids, assembloids have recently emerged as powerful in vitro tools for gaining deeper insight into human development and disease.^28^ Furthermore, for in vivo regeneration of tissue defects, the ability of cells to remain as aggregates within spheroids or to migrate into the defect space is an important factor in determining the regenerative outcome.^29^ Despite the increasing evidence regarding the influence of matrix viscoelasticity on cellular behavior,^9^, ^17^ the effect of hydrogel viscoelasticity on cell migration for spheroid fusion has not been investigated.

Moreover, growth factors such as platelet‐derived growth factor (PDGF) provide potent biochemical cues that can activate migration of various cell types including MSCs.^30^ Clinically, recombinant human PDGF‐BB is one of only two growth factors approved by the US Food and Drug Administration (FDA) for clinical application in craniofacial regenerative medicine and can act by recruiting endogenous MSCs to a defect site.^31^, ^32^ However, the interplay between biochemical and biophysical cues on spheroid behavior remains largely unknown.

Here, we investigate the role of hydrogel viscoelasticity on cell migration and fusion of MSC spheroids and elucidate the interplay between the effects of matrix stress relaxation and PDGF on spheroid behavior. We hypothesize that fast stress relaxation behavior would facilitate the fusion of MSC spheroids, and PDGF would further enhance this phenomenon. Alginate‐based hydrogels have been previously shown to enable decoupling of the elastic response from the viscous behavior of hydrogels.^11^, ^14^ Two alginate hydrogel compositions of similar elasticity, but different stress relaxation behavior were used to explore this hypothesis. Murine MSCs were encapsulated in these hydrogels to study changes in spheroid area and fusion over time. Rho‐associated protein kinase (ROCK) and Rac family small GTPase 1 (Rac1) inhibitors were subsequently used to block actomyosin contraction and actin polymerization in cells, to probe their role. Finally, to evaluate the broader clinical applicability of these findings, spheroids composed of human bone marrow‐derived MSCs (hBM‐MSCs) and human dental pulp stem cells (hDPSCs) were studied.

## 2 MATERIALS AND METHODS

### 2.1 Hydrogel preparation

Sodium alginate (Protanal® LF 10/60) with an average molecular weight (MW) of 145 ± 11 kDa was obtained from Dupont Nutrition & Biosciences (previously known as FMC BioPolymer) and used as the high MW (HMW) alginate for hydrogel preparation. To prepare alginate chains with a lower MW (LMW), this HMW alginate was gamma irradiated at a dose of 5 Mrad using a Cobalt source, resulting in an average MW of 39 ± 2 kDa. Average MW of alginates was determined using gel permeation chromatography, as reported previously.^33^

Modification of alginate chains with arginine–glycine–aspartate (RGD) ligands was carried out through covalent coupling of GGGGRGDSP peptides to alginate chains based on carbodiimide chemistry using *N*‐hydroxysulfosuccinimide (Sulfo‐NHS; Thermo Scientific Pierce) and *N*‐(3‐dimethylaminopropyl)‐*N*′‐ethylcarbodiimide hydrochloride (EDC; Sigma‐Aldrich) as reported previously.^34^, ^35^ Using this strategy, ~20 RGD motifs were coupled to each HMW alginate chain. For the RGD modification of LMW alginate chains, the RGD content per mass of alginate was kept the same as that for the HMW alginate (~145 μmol/g). Thereafter, RGD‐modified alginate was dialyzed for 3–4 days against decreasing concentrations of aqueous sodium chloride solutions (7.5–0.0 g/L) using regenerated cellulose dialysis tubing (Spectrum Laboratories, Inc.) with a MW cutoff of 3.5  kDa. Thereafter, the alginate solutions were treated with activated charcoal (Sigma‐Aldrich), vacuum sterile filtered (0.22 μm pore‐size filters), lyophilized, and stored in dry form at −20°C until further usage.

Prior to hydrogel formation, alginates were dissolved in serum‐free Dulbecco's Modified Eagles Medium (DMEM; Gibco). To prepare 2% w/v alginate gels, 0.8 ml of an alginate solution composed of 2.5% w/v LMW/HMW alginate was loaded into a 3 ml syringe. At the same time, 0.2 ml of serum‐free DMEM containing 91.5 mM (for HMW alginate) or 183.0 mM (for LMW alginate) of calcium sulfate was loaded into another 3 ml syringe. After removing air bubbles from the syringes by manual tapping, the two syringes were connected using a female–female Luer‐lock connector. Thereafter, the contents of the two syringes were mixed rapidly to initiate the gelation process, and the mixture was immediately deposited on a surface for gelation.

A two‐step casting process was employed based on the modification of a previously reported methodology for the encapsulation of multicellular spheroids within a single geometric plane in order to facilitate their confocal imaging and quantitative analyses.^36^ For this process, standard 6‐well plates were used, and plastic inserts (Figure S1A) were fabricated using an Objet30 3D Printer (Stratasys) based on the VeroBlue material (Stratasys). As illustrated in Figure S2, the first alginate layer (1 mm thick) was cast by deposition of the alginate mixture into a well and immediate placement of an insert. The mixture was then maintained for an hour at room temperature to fully gelate. Thereafter, the insert was removed from the well, and a sterile gauze pad was delicately placed on the top surface of the alginate disk. An aqueous solution containing 100 mM of sodium citrate (Sigma‐Aldrich) and 30 mM of ethylenediaminetetraacetic acid disodium salt (EDTA; Sigma‐Aldrich) was sterile filtered and was added dropwise onto the gauze surface to wet the whole pad without an excess. After 2 min, the pad was gently removed from the gel surface. This process results in the availability of uncrosslinked alginate chains at the gel surface to achieve full and uniform bonding with the second layer. 100 μl of 2% w/v alginate (with or without spheroids) were then uniformly distributed onto the gel surface. Prior to casting the second gel layer, a 1 mm thick ring (Figure S1B) was placed into the insert to allow for a gap height of 2 mm inside the wells (instead of 1 mm gap used for the first layer). Thereafter, the second alginate layer (1 mm thick) was cast by depositing an alginate mixture into the well, immediately followed by the placement of a height‐adjusted insert into the well. To ensure uniform ionic cross‐linking of the alginate construct, serum‐free DMEM containing a calcium concentration similar to that of the gel was added into the well. The plate was incubated for 30 min in a cell culture incubator (Figure S2). Thereafter, the insert and medium were removed, and alginate disks (8 mm diameter and 2 mm thickness) were obtained by punching the constructs using an 8 mm biopsy punch.

### 2.2 Rheological test

Viscoelastic properties of hydrogels were evaluated with a Discovery Hybrid Rheometer (TA instruments) using a parallel plate stainless‐steel geometry (diameter = 20 mm). Immediately after mixing the alginate solution with the calcium sulfate dispersion, the mixture was deposited onto the Peltier plate of the rheometer and the gap height was set to 1 mm. Low viscosity silicon oil was used to seal the gap to minimize hydrogel drying during the rheological tests. A time‐sweep was then performed at 25°C for 1 h at 1% strain and 1 Hz frequency to allow for complete gelation of the sample. The temperature was then increased to 37°C and another time sweep (1% Strain and 1 Hz) was performed for 15 min to ensure temperature equilibration. The final time point of this step was used to determine the storage moduli of the hydrogels. Afterward, a stress relaxation experiment was carried out at 37°C by applying 15% strain and monitoring the generated stress for 3 h. The stress relaxation data were normalized to the stress recorded at 0.1 s for each measurement. The stress relaxation halftime (𝜏_1/2_) was determined as the time at which the initial stress decayed to half of its initial value (at 0.1 s).

### 2.3 Nanoindentation

A G200 nanoindenter (Keysight Technologies) was used to evaluate the viscoelastic properties of cross sections of hydrogels (*n* = 4) prepared using the two‐step casting process. Hydrogel cross sections were prepared by sectioning each sample using a surgical blade. The cross sections were transferred to glass slides for performing the indentation tests, and droplets of cell culture medium were added around the cross sectioned samples to minimize drying. Dynamic indentations were carried out at three locations of the cross section of each sample: middle region (0.0 mm from midline), middle of first casted layer (−0.5 mm from midline), and middle of second casted layer (+0.5 mm from midline) (Figure S3). The tests were carried out in air at room temperature using an oscillation frequency and amplitude of 110 Hz and 500 nm, respectively. Each indentation was performed at an indentation depth of 4.5 ± 0.2 μm using a spherical indenter with a diameter of 400 μm.

### 2.4 Cell culture

Mouse bone marrow stromal‐derived MSCs were obtained from a commercial supplier (D1 MSC, CRL‐12424, ATCC). The cells were cultured in high‐glucose GlutaMAX Dulbecco's Modified Eagles Medium (DMEM, ThermoFisher) with 10% fetal bovine serum (FBS) and 1% penicillin/streptomycin (P/S). The cells were cultured at sub‐confluency (70% maximum) to maintain stemness, passaged (up to P10 maximum), and medium was changed every 2 days.

Similarly, human bone marrow‐derived MSCs (hBM‐MSCs, PCS‐500‐012, ATCC) and human dental pulp stem cells (hDPSCs, PT‐3927, Lonza) were obtained from commercial suppliers and cultured using established protocols with culture medium purchased from the respective suppliers. hBM‐MSCs were cultured in MSC basal medium (PCS‐500‐030, ATCC) supplemented with 7% FBS, rh IGF‐1 (15 ng/ml), rh FGF‐b (125 pg/ml), l‐alanyl‐l‐hlutamine (2.4 mM), and 1% P/S. hDPSCs were cultured using a DPSC BulletKit (PT‐3928 and PT4516, Lonza). Both primary human cell types were cultured at sub‐confluency (maximum 70%) to maintain stemness, passaged (up to P5 maximum), and medium was changed every 2 days.

### 2.5 Spheroid formation

Spheroids of controlled size and morphology were generated based on a forced aggregation technique using AggreWell™ 400 6‐well plates (STEMCELL Technologies). Cells were detached from culture flasks using 0.05% trypsin EDTA and collected by centrifugation at 1200 rpm for 5 min. AggreWell plates were consequently treated with an anti‐adherence rinsing solution (STEMCELL Technologies), warm basal medium, and then complete medium. 5.90 × 10^6^ or 2.95 × 10^6^ cells in 5 ml of medium were seeded into each well to achieve a cell density of about 1000 or 500 cells per D1 MSC/hDPSC or hBM‐MSC spheroid, respectively. After cell seeding, the plates were centrifuged at 100 g for 3 min to achieve the forced aggregation of cells into the wells. The plates were next incubated overnight at 37°C with 5% CO_2_ to allow for spheroid formation. This short incubation period was chosen to ensure sufficient nutrients for cell viability in the AggreWell plates and to avoid the need for refreshing the medium, which could disrupt spheroids localization in microwells. Subsequently, spheroids were harvested by pipetting the medium gently to resuspend the spheroids from the microwells. Harvested spheroids were collected into 50 ml Falcon tubes, centrifuged at 100 g for 3 min, and resuspended in complete DMEM.

### 2.6 Spheroid encapsulation

For migration and fusion studies, spheroid encapsulation was carried out using the two‐step casting process described in Section 2.1. To this end, spheroids were dispersed in 2% w/v alginate solutions, which were applied after casting the first alginate layer (Figure S2). Each punched hydrogel disk (8 mm diameter and 2 mm thickness) contained ~350 spheroids, which were randomly distributed throughout its middle plane. Due to the consistent dimensions of the microwells employed for spheroid formation, spheroids made of each cell type were of similar size and mass. Therefore, despite the influence of gravity during spheroid culture, the encapsulated spheroids in different hydrogel compositions can largely remain within a geometrical plane (Figure S4). For cell proliferation studies using DNA assay, spheroid encapsulation was carried out in a one‐step casting process as described previously (Figure S5).^14^ Hydrogel disks (8 mm diameter and 1 mm thick) were obtained using a biopsy punch, each of which contained ~350 spheroids randomly distributed throughout their matrix.

### 2.7 Spheroid culture

Following spheroid encapsulation, hydrogel disks were transferred to 24‐well plates where they were immersed in 1 ml of corresponding complete growth medium for each cell type and cultured for up to 5 days. To assess the effect of biochemical cues on MSC migration for spheroid fusion, mouse (rmPDGF‐BB) or human (rhPDGF‐BB) platelet‐derived growth factors were included in the cell culture media at a concentration of 10 ng/ml. The growth factor supplemented media were refreshed every 48 h.

To elucidate the cellular mechanism involved in 3D MSC migration for spheroid fusion, small‐molecule inhibitors were employed following previous literature.^14^ For these experiments, 10 μM of Y‐27632 (ATCC) was used to inhibit ROCK and block actomyosin contraction, and 50 μM of NSC‐23766 (Selleckchem) was used to inhibit Rac1 and block actin polymerization. These inhibitors were included in the media used for the culture of encapsulated spheroids, which were refreshed every 48 h.

### 2.8 Spheroid fixation and immunocytochemistry

Hydrogels containing MSC spheroids were rinsed three times with PBS containing 10 mM calcium (cPBS) and fixed in 4% paraformaldehyde (PFA) in cPBS for 30 min, treated with EDTA for 15 min, and permeabilized in 0.5% Triton X‐100 in 3% goat serum overnight. Phalloidin‐Alexa Fluor™ 488 (AF488, Life Technologies) at a dilution of  1:100 was added to stain the actin cytoskeleton overnight. After rinsing with PBS, Hoechst at a dilution of 1:1000 was added to stain nuclei. Gels were transferred to microscope slides and Prolong Gold antifade reagent (Invitrogen) was added and were maintained at room temperature overnight.

### 2.9 Confocal microscopy

Confocal fluorescent microscopy was carried out using an upright LSM 710 confocal microscope (Zeiss) and involved imaging of Phalloidin‐AF488 and Hoechst channels. For quantification of spheroid area and fusion, a 4x air objective was used to perform tile scans of complete area of each sample. These scans involved Z‐stacks images from the middle region of each disk where spheroids were located. Subsequently, images were processed for further analysis via maximum intensity projection of the z‐stacks of each tile scan. For visualization purposes, higher magnification images were acquired using a 10x air objective.

### 2.10 Quantification of spheroid area and fusion

Analyses of spheroid area, spheroid fusion, and inter‐spheroid distance were carried out using CellProfiler™ Software.^37^ Prior to the analysis, ImageJ software was employed to convert fluorescent images to 8‐bit greyscale format. Thereafter, to enhance the accuracy of the automatic quantification using CellProfiler, "Brush Tool" was employed in ImageJ to manually annotate the center of each spheroid, and "Selection Tools" were used to exclude high brightness defects from samples. Next, a CellProfiler Pipeline was utilized to analyze each image involving “identification of primary objects” (i.e., spheroid cores) and “identification of secondary objects” (i.e., total area of each spheroid including migrated cells). For the “identification of secondary objects,” a “propagation” method was employed in the CellProfiler settings to define the boundaries between outgrowth area of different spheroids. In this method, the boundary lines are determined based on distance to the spheroid cores (primary objects) and intensity gradients of the outgrowth area. More specifically, the “propagation” algorithm identifies the boundaries based on local image similarity and the boundary lines are positioned at locations where the local appearance in the image varies perpendicularly to the boundary line.^38^ Accordingly, area, distance to closest neighbor, total number of neighboring spheroids (fused + nonfused), and number of adjacent spheroids (fused) were extracted for each spheroid. Average diameter (D_avg_) of spheroids upon formation was calculated using the area of spheroids at Day 0 assuming full circularity. Inter‐spheroid distances were determined from the center of spheroids (cores). As illustrated in Figure S6, inter‐spheroid fusion (%) was quantified for spheroids positioned within D_avg_ < inter‐spheroid distance < 2 × D_avg_ of their closest neighbor, using the following equation:

$$\text{Inter‐spheroid fusion}\;\left(\%\right)=\frac{\text{Number of adjacent spheroids}}{\text{Total number of neighboring spheroids}}\times 100.$$

### 2.11 Cell proliferation

Cell proliferation of spheroids cultured in hydrogels was evaluated using a Quant‐iT Pico Green dsDNA Assay Kit (Thermo Fisher Scientific, USA). After the culture period, hydrogel disks containing D1 MSC spheroids were rinsed three times with DNA/RNAse/Protease‐free water (Growcells, USA), placed in individual tubes, minced with surgical blade and diluted in 1x Passive Lysis Buffer (Promega, USA). The samples were sonicated for 20 s and centrifuged at 12,000 g for 15 s. The supernatant was transferred to a new tube. Total DNA content from each sample was determined according to the assay manufacturer instructions (Figure S5).

### 2.12 Statistics

Statistical analyses were performed using GraphPad Prism 9 software. Statistical comparisons among experimental conditions for spheroids studies were carried out using analysis of variance (ANOVA) tests. Equality of variance for these tests was evaluated using Bartlett's and Brown–Forsythe homoscedasticity tests. Brown–Forsythe and Welch ANOVA tests, followed by Games–Howell's multiple comparisons test, were employed for analyses of spheroid migration and fusion results, as these datasets did not exhibit equal variances. Two‐way ANOVA, followed by Tukey's multiple comparisons test, was employed for analysis of DNA assay results, as these datasets exhibited equal variances. Given the large sample size employed in this study for spheroid migration and fusion analyses (51–1333 spheroids per group), data normality was assumed based on the central limit theorem. The DNA assay data passed the Shapiro–Wilk test for normality. Statistical comparisons among experimental conditions for rheological and nanoindentation results were made using an unpaired *t*‐test. Results for each experimental condition were obtained from three to four biologically independent experiments.

## 3 RESULTS AND DISCUSSION

### 3.1 Tuning viscoelasticity of hydrogels

**FIGURE 1:**
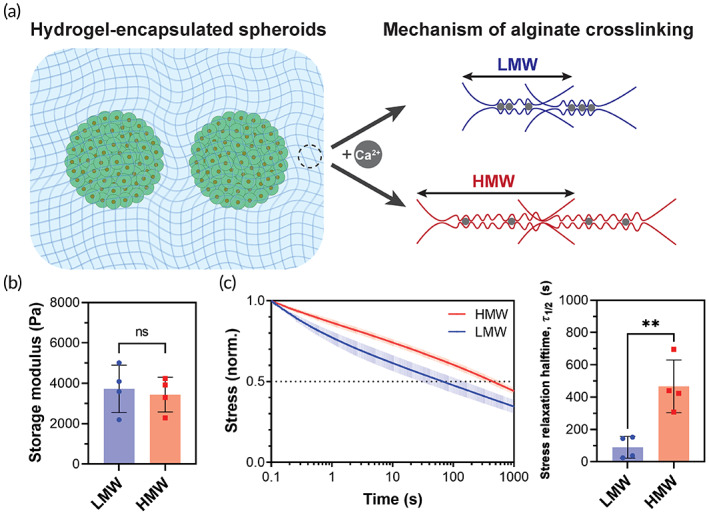
Hydrogel matrices for spheroid encapsulation. (a) Schematic illustration of crosslinking mechanism of hydrogels made of LMW or HMW alginate. (b) Storage moduli and (c) stress relaxation behavior of hydrogel compositions. ** and ns indicate *p* ≤ 0.01 and statistically not significant (*p* > 0.05), respectively. Data are shown in (b, c) as mean ± s.d. for *n* = 4 independent tests per group.

Alginate cross‐linking was achieved by the introduction of Ca^2+^ ions, which can interact with the guluronate block of alginate through noncovalent ionic interactions (Figure 1a). Upon the introduction of 36.6 or 18.3 mM Ca^2+^ solutions in 2% w/v solutions of LMW or HMW alginate, hydrogels were formed with average storage moduli (*G*′) of 3.7 ± 1.2 or 3.4 ± 0.9 kPa (Figure 1b), respectively. *G*′ values of the two hydrogel compositions exhibited no statistical difference, indicating a similar elastic response for the LMW and HMW hydrogels formulated in this study. In contrast, when subjected to a constant strain, the stress generated in these hydrogels decayed with different relaxation profiles (Figure 1c). Quantification of the stress relaxation halftime (𝜏_1/2_) revealed an average 𝜏_1/2_ of 89 ± 68 s or 467 ± 164 s for the LMW or HMW hydrogels, respectively. Given these stress relaxation profiles, hereafter, we refer to the LMW or HMW hydrogel compositions as FR or slow relaxing (SR) hydrogels, respectively.

Hydrogel materials can undergo swelling and degradation upon exposure to biological fluids, thereby changing their mechanical properties.^39^ Nevertheless, the alginate‐based hydrogel system employed in this work has been shown in several previous investigations to be mechanically stable, displaying negligible degradation and swelling when incubated in cell culture media for 3 weeks.^35^

To enable facile confocal imaging of spheroids and quantification of inter‐spheroid distances in 2D images, a two‐step hydrogel casting methodology was used to encapsulate spheroids within a single geometrical plane. Oscillatory nanoindentation tests confirmed that hydrogel viscoelasticity was uniform across the cross section of the hydrogel disks (Figure S3).

### 3.2 Migration behavior of spheroids encapsulated within viscoelastic hydrogels

**FIGURE 2:**
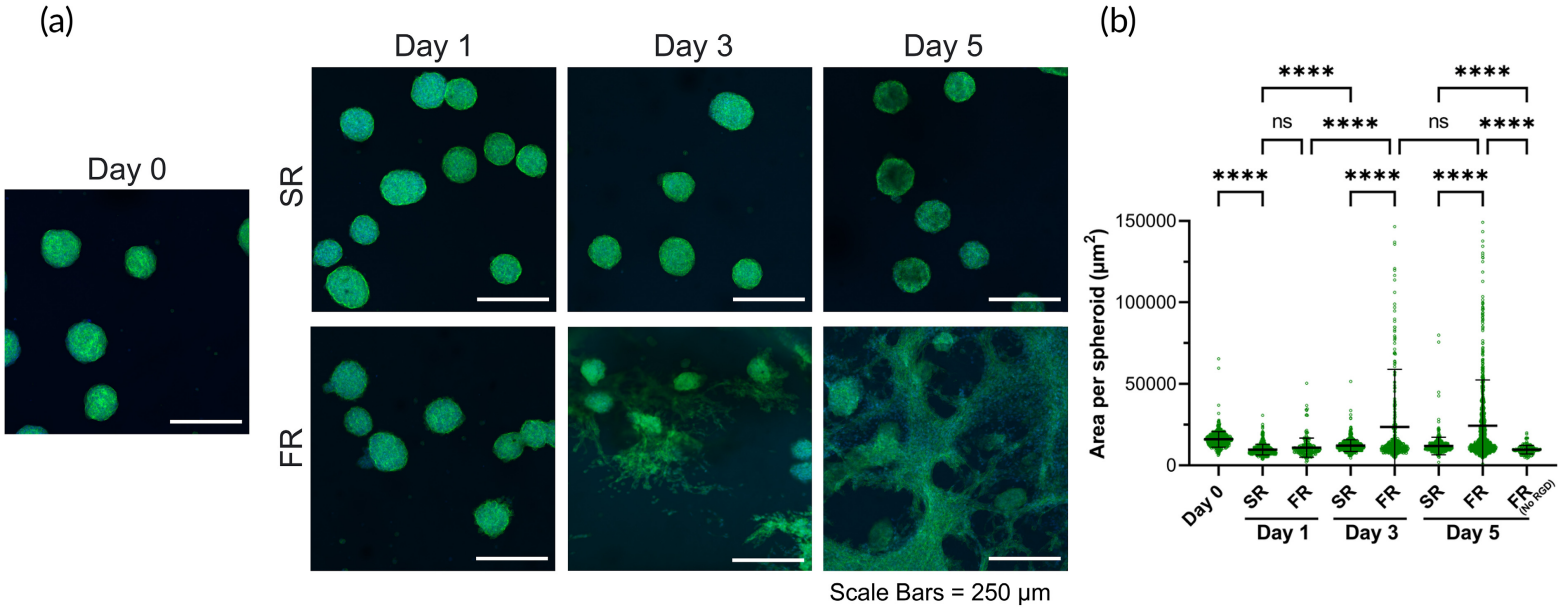
Cell migration from MSC spheroids as function of hydrogel viscoelasticity. (a) Confocal images of spheroids cultured over time in hydrogels without inclusion of PDGF‐BB. Green = actin cytoskeleton. Blue = nucleus. (b) Area per spheroid in SR or FR hydrogels. Spheroids consisted of mouse bone marrow‐derived MSCs. **** and ns indicate *p* ≤ 0.0001 and statistically not significant (*p* > 0.05), respectively; Brown–Forsythe and Welch ANOVA tests, followed by Games–Howell's multiple comparisons test. Data points represent individual spheroids, based on *n* = 161–939 spheroids analyzed per group from three to four biologically independent experiments.

MSC spheroids were formed and encapsulated in FR or SR hydrogels, and area per spheroid (μm^2^) was analyzed as a marker for cell migration from the spheroids across a 5‐day time course (Figure 2a). The spheroids cultured in FR gels exhibited significantly higher increase in area as compared to those cultured in SR gels over 5 days (Figure 2b). The largest increase in area occurred in FR gels from Day 1 to Day 3. However, comparing Day 3 to Day 5 for this group, there was no significant differences in area suggesting that most cell migration and spheroid spreading occurred between Day 1 and Day 3. Interestingly, in both FR and SR gels, spheroids showed a decrease in area upon 1 day of culture, suggesting spheroids underwent further contraction at an early stage of culture in the hydrogels. Such spheroid compactions are commonly observed during spheroid formation when employing non cell‐adhesive biomaterials, arising from favored cell–cell over cell–matrix adhesive interactions via homophilic cadherin–cadherin bindings.^40^ Indeed, in the absence of RGD ligands, spheroid compaction was evident and cell migration and spheroid spreading did not occur even in FR gels (Figure 2b). This observation indicates that matrix viscoelasticity can play a similarly significant role as cell adhesive ligands for engineering spheroid behavior. The maximum effective degree of RGD modification of HMW alginates (MW of 100–350 kDa) was previously found to be within a range of 20–30 RGD motifs per alginate chain, which is consistent with the degree of RGD modification in this study (i.e., 20 RGDs/chain for HMW alginate chain).^41^ Previous studies suggest that a higher modification degree of alginate polymers would result in steric hindrance of cell binding as a result of RGD spacings smaller than the diameter of integrin receptors (~10 nm).^41^, ^42^, ^43^ Interestingly, for RGD spacings larger than 30 nm, 2D migration studies for MSCs and endothelial cells have shown a nonmonotonic change of migration speed as a function of RGD spacings.^44^, ^45^ Indeed, a previous study found a greater degree of cell migration when MSC spheroids were encapsulated in alginate gels with a lower degree of RGD modification.^46^ Accordingly, lower RGD modification degrees of alginate hydrogels might result in such a nonmonotonic response in 3D MSC migration and spheroid fusion, which requires further investigation.

**FIGURE 3:**
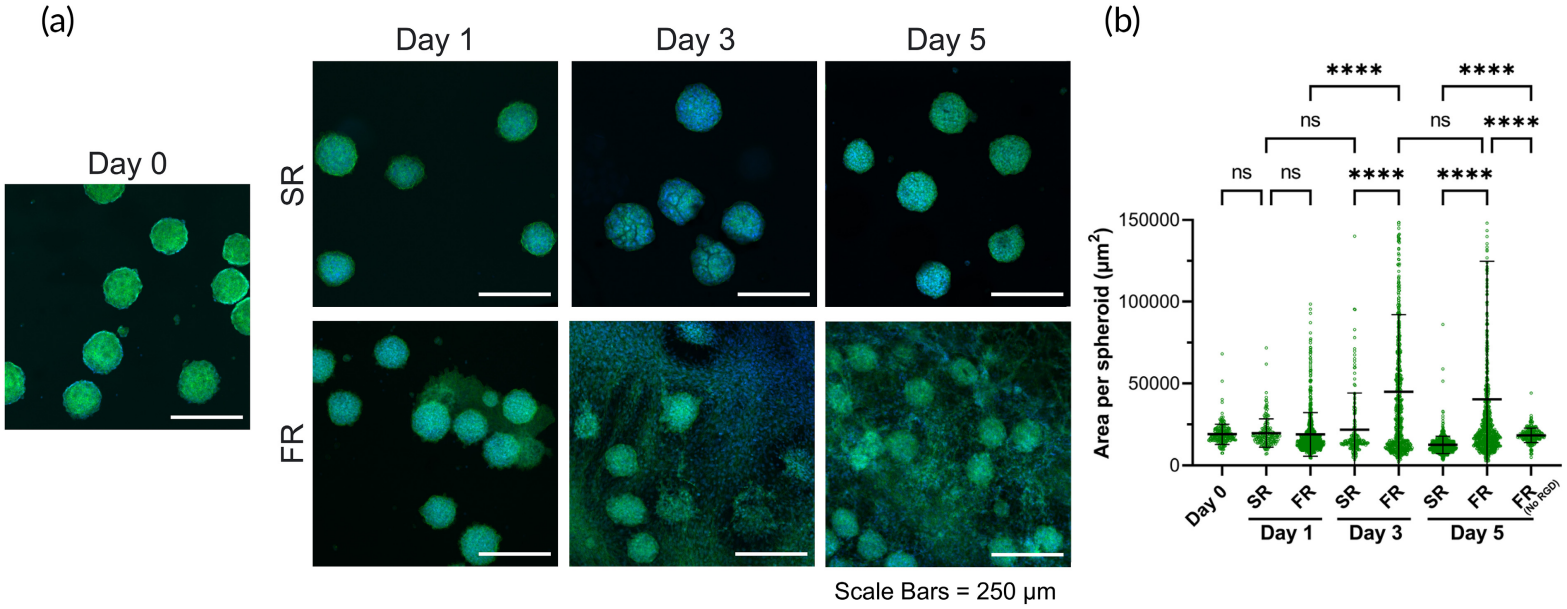
Cell migration from MSC spheroids upon inclusion of PDGF‐BB. (a) Confocal images of spheroids cultured over time in hydrogels using growth media supplemented with rm‐PDGF‐BB (10 ng/ml). Green = actin cytoskeleton. Blue = nucleus. (b) Area per spheroid in SR or FR hydrogels for samples cultured in growth media supplemented with rm‐PDGF‐BB (10 ng/ml). Spheroids consisted of mouse bone marrow‐derived MSCs. **** and ns indicate *p* ≤ 0.0001 and statistically not significant (*p* > 0.05), respectively; Brown–Forsythe and Welch ANOVA tests, followed by Games‐Howell's multiple comparisons test. Data points represent individual spheroids, based on *n* = 178–804 spheroids analyzed per group from three to four biologically independent experiments.

To determine whether biochemical signals can further influence this migratory behavior in addition to biophysical cues, PDGF‐BB, a potent activator of MSC migration,^27^ was introduced to the culture media used for spheroid culture (Figure 3a). The area of spheroids encapsulated in SR gels did not undergo a statistically significant increase throughout the 5‐day time course despite the addition of PDGF. In contrast, spheroids encapsulated in FR gels exhibited a significant increase in area throughout the time course compared to Day 0 (Figure 3b). In the presence of PDGF, the largest increase in area occurred in FR gels from Day 1 to Day 3. In addition, comparing Day 3 to Day 5, there was no significant differences in area suggesting that most cell migration and spheroid spreading occurred between Day 1 and Day 3, similar to when spheroids were cultured in growth media without PDGF supplementation. Interestingly, in both FR and SR gels at Day 1, the area per spheroid did not undergo significant changes in the presence of PDGF, in contrast to shrinkage in the absence of PDGF. PDGF supplementation alone could not compensate the impact of viscoelasticity or RGD ligands, as evident by the low area of PDGF‐supplemented spheroids in SR or FR (No RGD) hydrogels at Day 5.

In addition, PDGF is a mitogen for MSCs, and therefore cell proliferation may be a contributing factor for the observed enhancement of spheroid area.^47^ To determine the potential impact of cell proliferation on these results, we cultured spheroids in the two hydrogel types in the presence or absence of PDGF for 5 days and quantified the total DNA content in each group as a measure of cell number. While there was a trend for greater DNA content in FR gels with and without PDGF, no statistically significant differences were found among these different groups (Figure S5).

Altogether, these results demonstrated that matrix viscoelasticity plays a critical role in MSC migration from spheroids. FR hydrogels were permissive to cell migration, in contrast to SR hydrogels that were restrictive. The addition of PDGF‐BB significantly enhanced MSC migration from and spreading of spheroids in FR matrices. Other growth factors may also stimulate MSC migration including fibroblast growth factor‐2 (FGF‐2), insulin‐like growth factor‐2 (IGF‐2), and stromal cell‐derived factor 1 (SDF‐1) but are not the subject of this study.^48^, ^49^ In addition, a previous study demonstrated that deletion of the PDGF receptor in MSCs leads to decreased migratory and mitogenic responses.^47^ Future studies will be required to assess the effects of various growth factors and their receptor blockade on MSC migratory potential and MSC spheroid fusion in viscoelastic matrices.

### 3.3 Fusion of spheroids encapsulated within viscoelastic hydrogels

**FIGURE 4:**
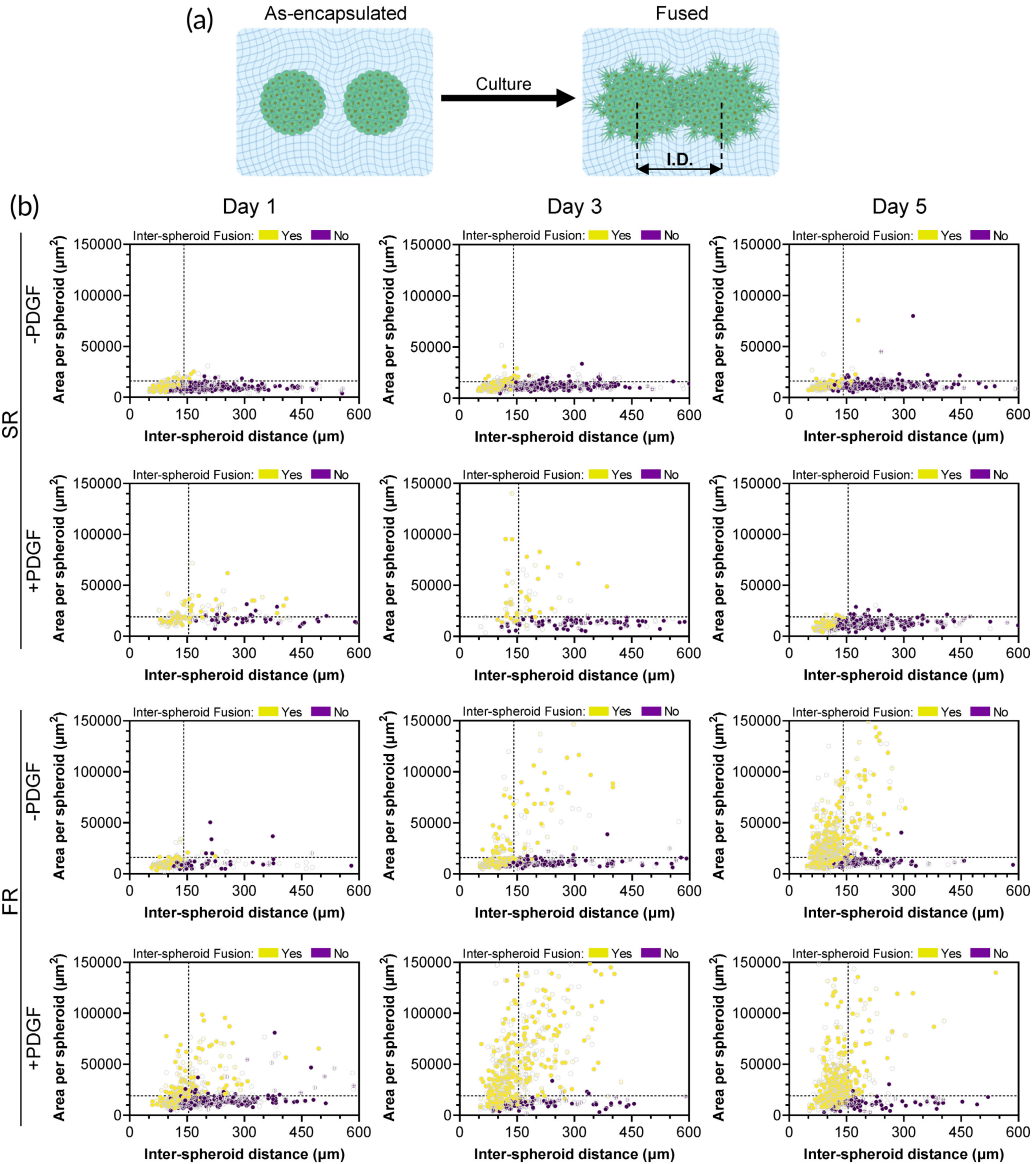
Spheroid behavior as function of inter‐spheroid distance. (a) Schematic illustration of fusion of spheroids at a specific inter‐spheroid distance (I.D.). (b) Scatter plots of spheroid area as a function of inter‐spheroid distance for spheroids encapsulated in slow relaxing (SR) or fast relaxing (FR) hydrogels cultured for up to 5 days in PDGF‐free (−PDGF) or PDGF‐supplemented (+PDGF) media. Horizontal and vertical dashed lines indicate the average spheroid area and the average inter‐spheroid distance of touching spheroids at Day 0, respectively. Yellow and purple dots indicate spheroids that were or were not in direct contact (fused) with at least one neighboring spheroid, respectively. All spheroids consisted of mouse bone marrow MSCs. Data points represent individual spheroids, based on *n* = 178–939 spheroids analyzed per group from three to four biologically independent experiments.

The fusion of spheroids with an initial inter‐spheroid gap would require bridging of proliferating and migrating cell populations (Figure 4a), and this bridging phenomenon is anticipated to be impacted by the gap size or distance between the spheroids, which is similar to in vitro wound healing assays.^50^ To gain a deeper insight into the fusion behavior of neighboring spheroids, the area of spheroids was next plotted, as well as their fusion status, as a function of their distance from the spheroid in closest proximity (i.e., inter‐spheroid distance) (Figure 4b). The average area of spheroids after formation (Day 0), and the average inter‐spheroid (center to center) distance of initially touching spheroids (i.e., average diameter) were denoted in these graphs as dashed lines to highlight the temporal changes of spheroids in different hydrogels and culture conditions relative to their initial characteristics. For the spheroids with inter‐spheroid distances of less than ~150 μm, fusion was largely evident for different hydrogels (FR or SR), culture conditions (without or with PDGF), or culture durations (up to 5 days), as these spheroids typically had initially touching neighbors. For the spheroids positioned at distances of more than ~150 μm (without initial direct contact), fusion behavior varied among different groups (Figure 4b).

For analysis of spheroid fusion, we have quantified inter‐spheroid fusion (%) for spheroids with an initial gap of up to ~150 μm with respect to their closest neighbor at Day 0. Previous studies suggest that the effective distance for paracrine signaling molecules to control cell migration and proliferation is approximately 250 μm.^27^ Here, the spheroids selected for analysis fall within the effective distance (250 μm) for spheroids crosstalk. However, as the spheroid distribution is random and similar among different groups, the potential role of paracrine signaling would not be expected to impact our conclusions.

**FIGURE 5:**
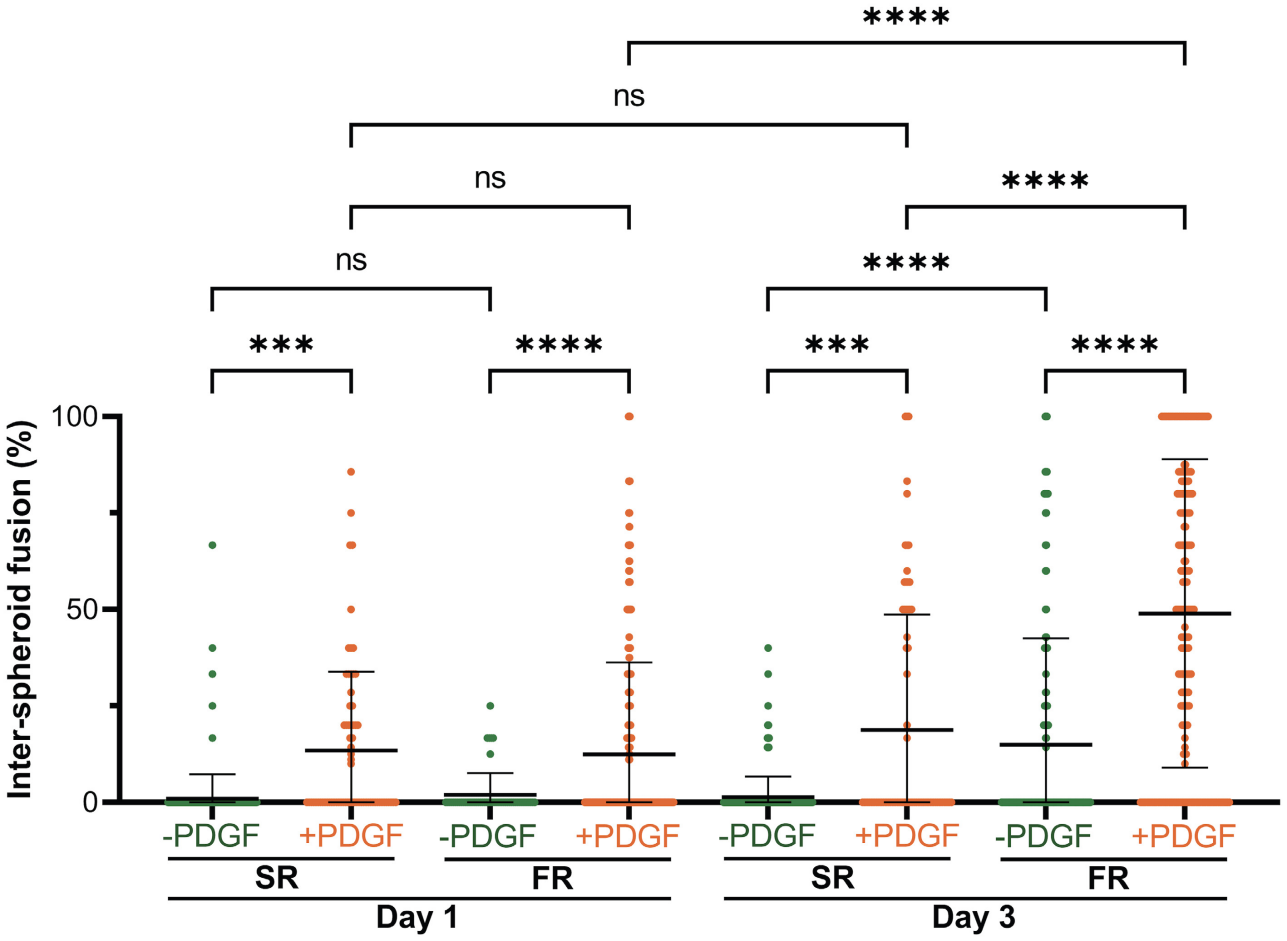
Quantification of inter‐spheroid fusion for spheroids encapsulated in SR or FR hydrogels cultured for 1 or 3 days in PDGF‐free (−PDGF) or PDGF‐supplemented (+PDGF) media. Values were obtained for spheroids positioned within D_avg_ < inter‐spheroid distance < 2 × D_avg_ of their closest neighbor, where D_avg_ is the average spheroid diameter at Day 0. ***, ****, and ns indicate *p* ≤ 0.001, *p* ≤ 0.0001, and statistically not significant (*p* > 0.05), respectively; Brown–Forsythe and Welch ANOVA tests, followed by Games–Howell's multiple comparisons test. Data points represent individual spheroids, based on *n* = 51–374 spheroids analyzed per group from three to four biologically independent experiments. All spheroids consisted of mouse bone marrow MSCs.

Overall, when comparing different experimental groups, a direct correlation was observed between spheroid area and the prevalence of inter‐spheroid fusion (Figure S7), indicating that the expansion of spheroid area upon cell migration allowed cells to bridge neighboring spheroids. For experimental groups supporting inter‐spheroid fusion, major fusion phenomena took place between Day 1 and Day 3 of the culture period (Figure 4b). Therefore, the degree of fusion at these two timepoints was quantified for the spheroids without initially touching neighbors and with at least one neighbor in their close vicinity (see Section 2.10 for details). These quantifications revealed that SR hydrogels alone did not support inter‐spheroid fusion, as only ~1% fusion was observed for this group at both timepoints (Figure 5). However, the supplementation of PDGF to spheroids encapsulated in SR hydrogels resulted in enhanced fusion at Day 1 (~13%). Nevertheless, this enhanced fusion did not further increase, as no significant difference was observed for the fusion % in SR (+PDGF) group between Day 1 and Day 3. FR hydrogels were able to support inter‐spheroid fusion without the need for PDGF supplementation, as the fusion % in FR (‐PDGF) group grew significantly from ~2% at Day 1 to ~15% at Day 3. Nonetheless, the highest degree of inter‐spheroid fusion occurred upon the combination of biophysical and biochemical cues, as spheroids encapsulated in FR hydrogels with PDGF supplementation displayed the highest fusion (~49%).

### 3.4 Role of Rac1 and ROCK

**FIGURE 6:**
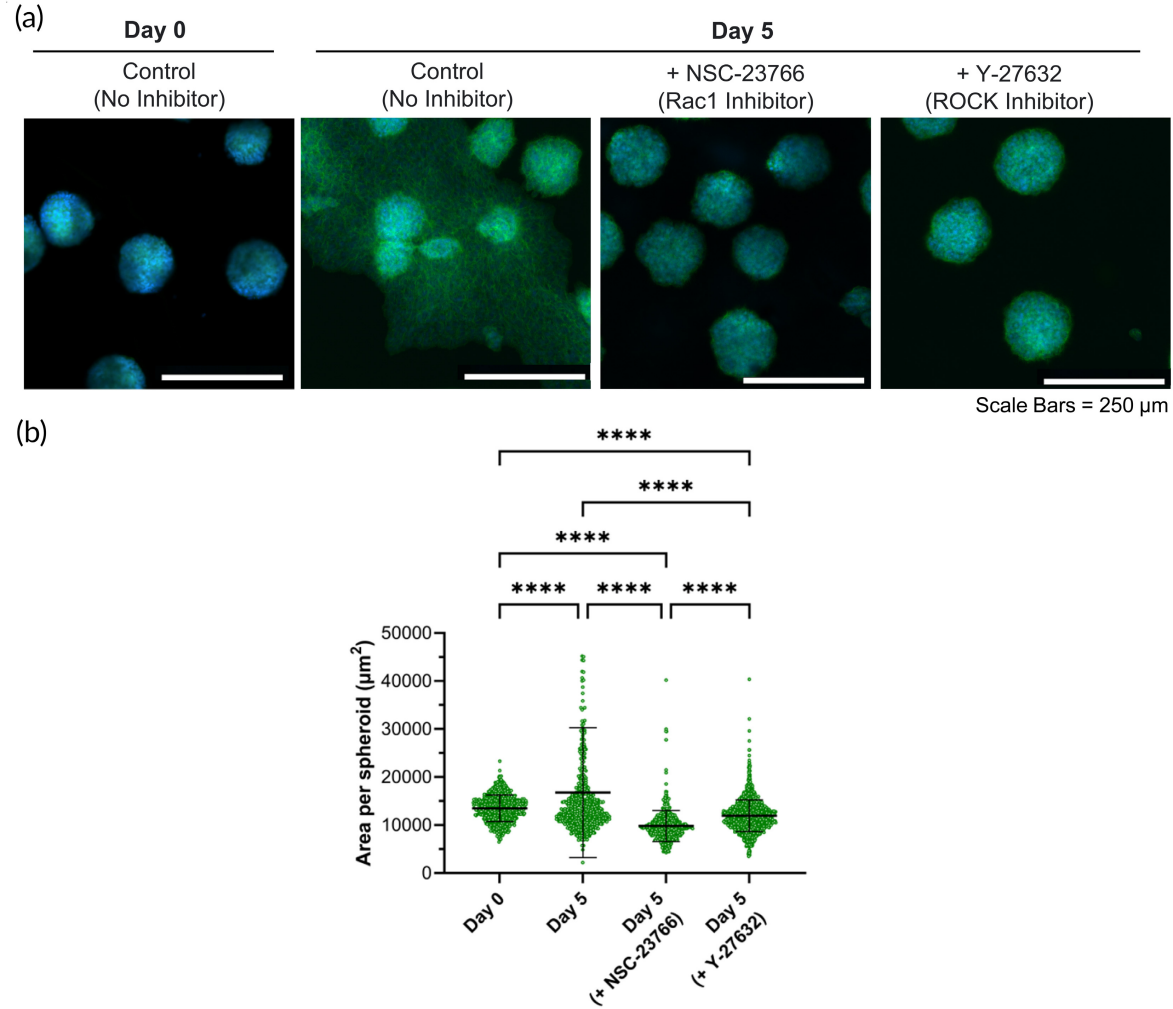
Role of Rac1 and ROCK. (a) Confocal images of MSC spheroids encapsulated in FR hydrogels cultured in growth medium supplemented with NSC‐23766 (Rac1 GTPase inhibitor) and Y‐27632 (ROCK inhibitor). Green = actin cytoskeleton. Blue = nucleus. (b) Area per spheroid cultured over time in FR hydrogels, without or with inhibitors. **** indicates *p* ≤ 0.0001; Brown–Forsythe and Welch ANOVA tests, followed by Games–Howell's multiple comparisons test. Data points represent individual spheroids, based on *n* = 563–1333 spheroids analyzed per group from three to four biologically independent experiments.

Cell motility is mediated by both actomyosin contraction at the rear of a migrating cell, and actin polymerization at the protruding edge, which involve ROCK and Rac1 pathways, respectively.^14^, ^51^ Accordingly, two small‐molecule inhibitors were next used to block actomyosin contraction (Y‐27632) and actin polymerization (NSC‐23766),^14^ to probe for mechanisms by which viscoelasticity impacts MSC migration (Figure 6a). Surface area per spheroid (μm^2^) was again used as a marker for cell migration from the spheroids (Figure 6b). Spheroids cultured in FR gels in medium without supplementation of the inhibitors exhibited a statistically significant increase in area at Day 5, as expected. In contrast, both FR hydrogel groups treated with inhibitors exhibited a decrease in spheroid area at Day 5, similar to spheroid behavior in restrictive SR gels or FR gels without RGD (Figure 2b). These data suggest that Rac1 GTPase and ROCK are key mediators of MSC migration from spheroids in viscoelastic hydrogels. These findings are in agreement with previous work in the mechanobiology assessing the role of matrix viscoelasticity on breast epithelial cells spheroids, MSC spheroids, and intestinal organoid behavior.^13^, ^14^, ^17^, ^19^ However, in contrast to previous findings in which ROCK inhibition did not have a significant effect on the breast epithelial cell (MCF10A) migration,^14^ our findings indicate that ROCK inhibition impacts MSC migration. Further mechanistic studies are needed to identify the molecular signaling pathways that regulate MSC migration in viscoelastic matrices.

### 3.5 Behavior of human MSC spheroids in viscoelastic hydrogels

**FIGURE 7:**
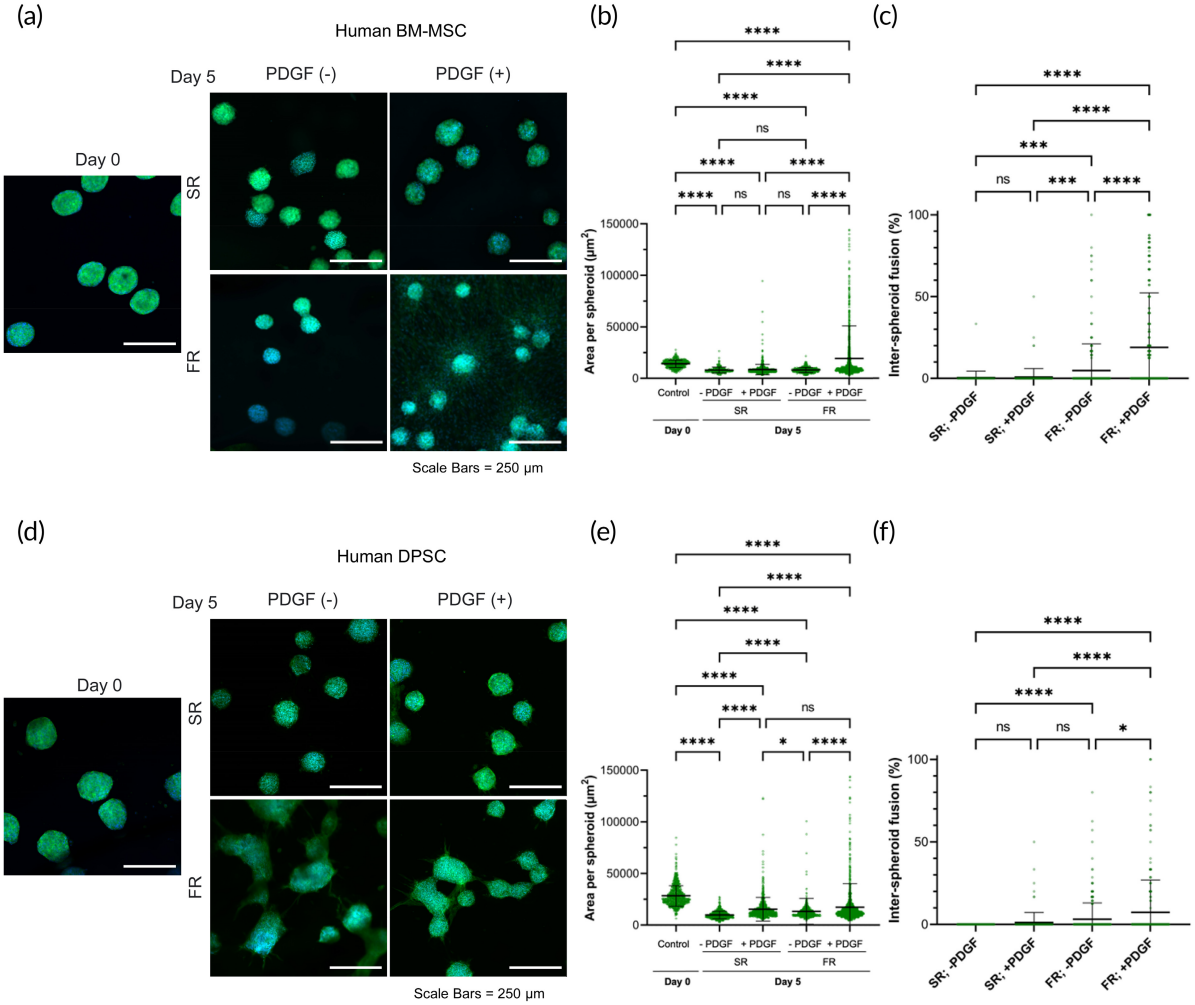
Migration and fusion behavior of human bone‐marrow and dental‐derived MSC spheroids. (a) Confocal images of human bone‐marrow‐derived MSC spheroids encapsulated in SR or FR hydrogels cultured in growth medium alone or supplemented with rh‐PDGF‐BB (10 ng/ml). Green = actin cytoskeleton. Blue = nucleus. (b) Area per spheroid and (c) inter‐spheroid fusion for hBM‐MSC spheroids cultured for 5 days in SR and FR gels with or without rhPDGF‐BB supplementation. (d) Confocal images of hDPSC spheroids encapsulated in SR or FR hydrogels cultured in growth medium alone or supplemented with rh‐PDGF‐BB (10 ng/ml). Green = actin cytoskeleton. Blue = nucleus. (e) Area per spheroid and (f) inter‐spheroid fusion for human dental pulp stem cells (hDPSC) spheroids cultured for 5 days in SR and FR gels with or without rhPDGF‐BB supplementation. *, ***, ****, and ns indicate *p* ≤ 0.05, *p* ≤ 0.001, *p* ≤ 0.0001, and statistically not significant (*p* > 0.05), respectively; Brown–Forsythe and Welch ANOVA tests, followed by Games–Howell's multiple comparisons test. Data points represent individual spheroids, based on (b) *n* = 215–1824, (c) *n* = 70–496, (e) *n* = 465–1254, (f) *n* = 132–280 spheroids analyzed per group from three to four biologically independent experiments.

As primary cells have higher variability when they are from different donors and exhibit phenotypic shifts with increasing passage number,^52^, ^53^ we performed initial studies mainly using a murine cell line to evaluate our hypothesis. Nevertheless, to verify whether the effect of matrix viscoelasticity on MSC migration and spheroid fusion is conserved across species and potentially applicable in humans, two primary human cell types, human bone marrow‐derived MSCs (hBM‐MSCs) and human dental pulp stem cells (hDPSCs), were used for spheroid formation and encapsulation in hydrogels (Figure 7). These human cells were chosen given their clinical relevance to demonstrate translational potential of our findings. hBM‐MSCs have been widely explored as a therapeutic cell type in many preclinical and clinical studies.^54^, ^55^, ^56^ In addition to hBM‐MSCs, hDPSCs were used as they are derived from a more accessible source, the oral cavity. MSC harvest from the bone marrow involves invasive surgical procedures such as bone marrow aspiration from the iliac crest, in contrast to harvesting from an oral source.^57^, ^58^ Thus, for future clinical application and reduction of morbidity associated with harvest, we investigate the use of a less invasive cell source.

For hBM‐MSCs, the area of spheroids encapsulated in SR gels did not increase with or without the presence of rhPDGF‐BB. In fact, spheroid shrinkage was observed at Day 5 for both conditions. In contrast, spheroids encapsulated in FR gels exhibited a significant increase in area only when supplemented with PDGF (Figure 7b). Similarly, spheroids encapsulated in SR gels did not undergo fusion despite PDGF supplementation. In comparison, spheroids encapsulated in FR gels displayed a significant increase in fusion when supplemented with PDGF **(**Figure 7c **)**.

The area of hDPSC spheroids encapsulated in SR gels did not increase with or without the presence of rhPDGF‐BB. Similar to hBM‐MSC spheroids, hDPSC spheroid shrinkage was observed at Day 5 for both conditions compared to Day 0. Surprisingly, spheroids encapsulated in FR gels and supplemented with rhPDGF‐BB also exhibited shrinkage compared to Day 0. This shrinkage behavior observed for different spheroid types in this study can be related to a continued compaction of cell clusters due to spheroid formation, as the spheroids were formed in microwells for only 24 h prior to encapsulation. Other investigations have reported that compaction for MSC spheroids can continue for 5 days during spheroid formation.^59^ Consequently, the incubation time of spheroids prior to their encapsulation in hydrogels can potentially impact the degree of shrinkage and area change observed in migration and fusion studies. Nevertheless, comparing between the conditions for hDPSC spheroids at Day 5, spheroids encapsulated in FR gels had higher area compared to those in SR gels with or without PDGF supplementation (Figure 7e). Spheroids encapsulated in SR gels exhibited negligible inter‐spheroid fusion despite PDGF supplementation. In comparison, spheroids encapsulated in FR gels displayed significantly higher fusion when supplemented with PDGF (Figure 7f).

Finally, when it comes to differences between hBM‐MSCs and hDPSCs, a previous study demonstrated that hDPSCs exhibited higher expression of E‐cadherin, and lower expression of Snail, an E‐cadherin repressor, than hBM‐MSC.^60^ Interestingly, an increase in human embryonic stem cell migratory capacity was associated with E‐cadherin downregulation.^61^ This could explain the difference between the migratory behavior between hBM‐MSC and hDPSC.

Together, these findings suggest potential differences in response to mechanical and biochemical cues of MSCs from different sources, and further studies will be needed to elucidate these mechanistic differences.

On a separate note, hydrogel viscoelasticity and PDGF supplementation are also capable of impacting a wide range of cell behaviors, including proliferation and differentiation, which might in turn affect migration and fusion behavior of spheroids.^11^, ^62^ While investigating these cellular behaviors were beyond the scope of this study, future studies will be needed to obtain a deeper understanding of the potential interplay between these factors. Additionally, while this study was focused on hydrogel compositions with a fixed storage modulus of ~3.5 kPa, it will be relevant to investigate in future studies the impact of matrix stress relaxation on spheroid behavior in hydrogels with lower or higher elasticities. More specifically, previous investigations have shown that hydrogels with elastic moduli of ∼17  kPa and a rapid stress relaxation are more favorable to osteogenic differentiation of MSCs.^11^ Therefore, for application of spheroid‐based systems in bone tissue regeneration, it will be relevant to study spheroid behavior in matrices with such high elasticities.

## 4 CONCLUSIONS

These findings demonstrate that matrix viscoelasticity plays an important role in the migration and fusion of spheroids formed from both mice and human MSCs, as spheroids encapsulated in matrices with permissive mechanical properties underwent enhanced migration and fusion. This effect can be further enhanced using PDGF as an activator of migration. Differences were observed in the extent of migration between MSCs from different sources (i.e., D1 MSC, hBM‐MSC, and hDPSC). Together, these results highlight matrix viscoelasticity as a key biophysical factor in the design of biomaterials for spheroid‐based strategies that are applicable in the development of in vitro tissue models and in vivo regenerative therapies. In particular, this finding not only provides a new biophysical avenue for controlling cellular behavior in spheroid‐based system but also emphasizes the potential hidden role of matrix viscoelasticity in other studies employing spheroids.

## AUTHOR CONTRIBUTIONS

**David Wu:** Conceptualization (equal); data curation (equal); formal analysis (equal); funding acquisition (equal); investigation (equal); methodology (equal); validation (equal); visualization (equal); writing – original draft (equal); writing – review and editing (equal). **Mani Diba:** Conceptualization (equal); data curation (equal); formal analysis (equal); funding acquisition (equal); investigation (equal); methodology (equal); supervision (equal); validation (equal); visualization (equal); writing – original draft (equal); writing – review and editing (equal). **Stephanie Yang:** Formal analysis (supporting); writing – review and editing (supporting). **Benjamin Freedman:** Conceptualization (supporting); formal analysis (supporting); methodology (supporting); writing – review and editing (supporting). **Alberto Elosegui‐Artola:** Conceptualization (supporting); investigation (supporting); methodology (supporting); writing – review and editing (supporting). **David Mooney:** Conceptualization (supporting); funding acquisition (lead); supervision (lead); writing – original draft (supporting); writing – review and editing (lead).

## CONFLICT OF INTEREST

The authors have no conflicts of interest to declare.

## Supporting information


**Figure S1.** Design of (A) insert and (B) ring employed for the two‐step casting of alginate gels.
**Figure S2.** Schematic illustration of the two‐step casting process employed for spheroid encapsulation.
**Figure S3.** (A) Schematic illustration showing the nanoindentation coordinates at a hydrogel cross section. (B) Storage moduli (*G*′), loss moduli (*G″*), and damping factor (tan(delta)) of FR hydrogels at different regions of the two‐step casted samples. NS indicates no statistically significant difference (*p* > 0.05).
**Figure S4.** Representative cross‐sectional views (y‐z) of confocal z‐stacks showing spheroids positioned within a plane after 5 days of culture within hydrogels made of high‐molecular weight (HMW) or low‐molecular weight (HMW) alginate. Scale bars = 500 μm.
**Figure S5.** Total DNA content in spheroids cultured under different conditions for 5 days. Total DNA content (ng/ml) was used as an indirect marker of the extent of cell proliferation. No statistically significant differences were detected between conditions; two‐way ANOVA followed by Tukey's multiple comparisons test was applied.
**Figure S6.** Overview of (A) the methodology used for quantification of inter‐spheroid fusion and (B) examples of fused and nonfused spheroids.
**Figure S7.** Total degree of fusion as function of average spheroid area for experimental groups cultured without (−PDGF) or with (+PDGF) supplementation. Each datapoint represents the mean value for an experimental group (compiled from three to four samples). Black continuous lines indicate the best‐fit line of simple linear regression. Outer dashed lines indicate 95% confidence bands.

## Data Availability

The data that support the findings of this study are available from the corresponding author upon reasonable request.
