# Supplementary material for: Hydrogel viscoelasticity modulates migration and fusion of mesenchymal stem cell spheroids
Source: Bioeng Transl Med. 2022 Dec 27;8(3):e10464. doi: 10.1002/btm2.10464 (PMC10189430; doi:10.1002/btm2.10464)
Supplement: Supplementary file 1 — Figure S1. Design of (A) insert and (B) ring employed for the two‐step casting of alginate gels. Figure S2. Schematic illustration of the two‐step casting process employed for spheroid encapsulation. Figure S3. (A) Schematic illustration showing the nanoindentation coordinates at a hydrogel cross section. (B) Storage moduli (G′), loss moduli (G″), and damping factor (tan(delta)) of FR hydrogels at different regions of the two‐step casted samples. NS indicates no statistically significant difference (p > 0.05). Figure S4. Representative cross‐sectional views (y‐z) of confocal z‐stacks showing spheroids positioned within a plane after 5 days of culture within hydrogels made of high‐molecular weight (HMW) or low‐molecular weight (HMW) alginate. Scale bars = 500 μm. Figure S5. Total DNA content in spheroids cultured under different conditions for 5 days. Total DNA content (ng/ml) was used as an indirect marker of the extent of cell proliferation. No statistically significant differences were detected between conditions; two‐way ANOVA followed by Tukey's multiple comparisons test was applied. Figure S6. Overview of (A) the methodology used for quantification of inter‐spheroid fusion and (B) examples of fused and nonfused spheroids. Figure S7. Total degree of fusion as function of average spheroid area for experimental groups cultured without (−PDGF) or with (+PDGF) supplementation. Each datapoint represents the mean value for an experimental group (compiled from three to four samples). Black continuous lines indicate the best‐fit line of simple linear regression. Outer dashed lines indicate 95% confidence bands. [file BTM2-8-e10464-s001.docx]

**Supporting Information**

**Hydrogel Viscoelasticity Modulates Migration and Fusion of MSC Spheroids**

David T. Wu^1,2,3,§^, Mani Diba^1,2,4,§^, Stephanie Yang^1,3^, Benjamin R. Freedman^1,2^, Alberto Elosegui-Artola^1,2,‡^, David J. Mooney^1,2,*^

^1^Laboratory for Cell and Tissue Engineering, John A. Paulson School of Engineering and Applied Sciences, Harvard University, Cambridge, MA, USA

^2^Wyss Institute for Biologically Inspired Engineering, Harvard University, Boston, MA, USA

^3^Department of Oral Medicine, Infection and Immunity, Harvard School of Dental Medicine, Boston, MA, USA

^4^Department of Dentistry-Regenerative Biomaterials, Radboud Institute for Molecular Life Sciences, Radboud University Medical Center, Philips van Leydenlaan 25, 6525 EX Nijmegen, the Netherlands

^§^These authors contributed equally to this work.

^‡^Current Address: Cell and Tissue Mechanobiology Laboratory, The Francis Crick Institute, and Physics Department, King’s College London, London, UK

^*^Corresponding author

Email address: [mooneyd@seas.harvard.edu](mailto:mooneyd@seas.harvard.edu)


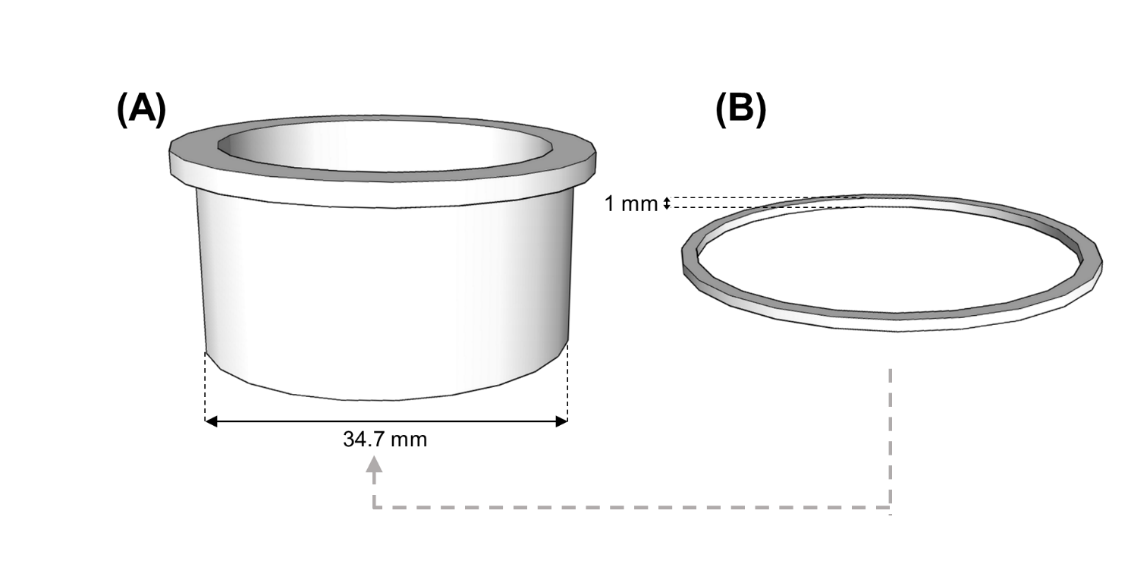


**Figure S1.** Design of (A) insert and (B) ring employed for the two-step casting of alginate gels.


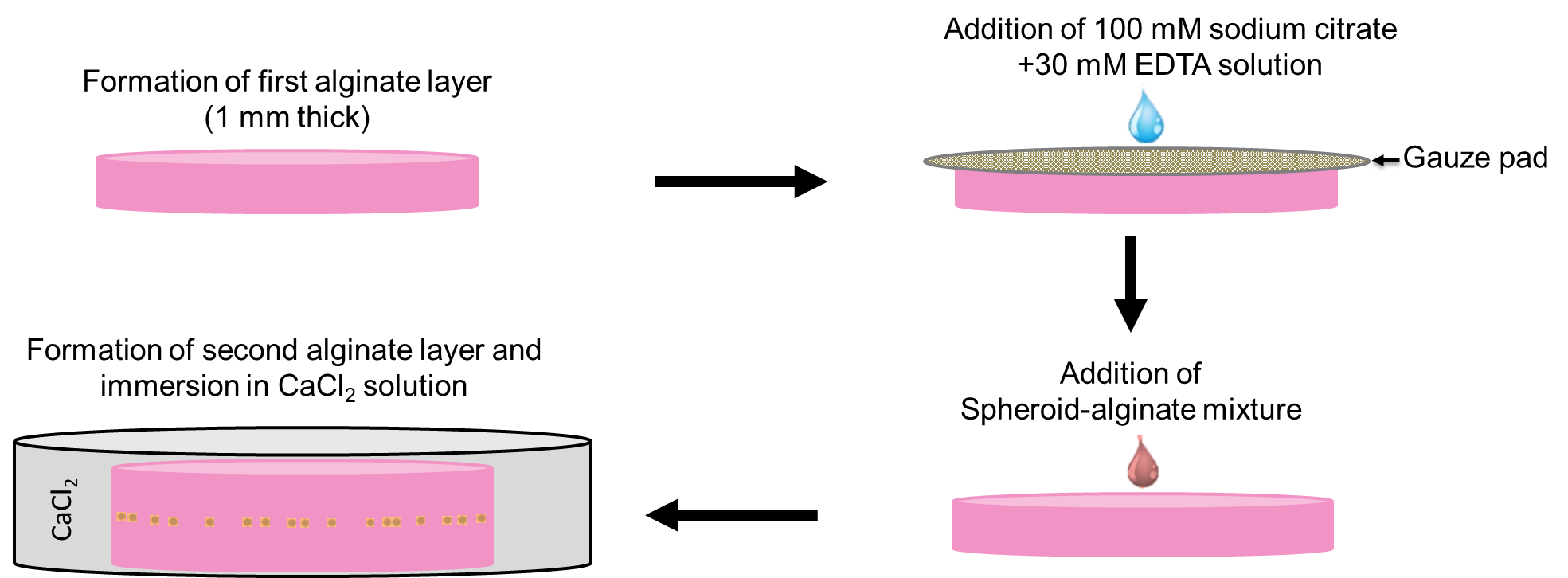


**Figure S2.** Schematic illustration of the two-step casting process employed for spheroid encapsulation.


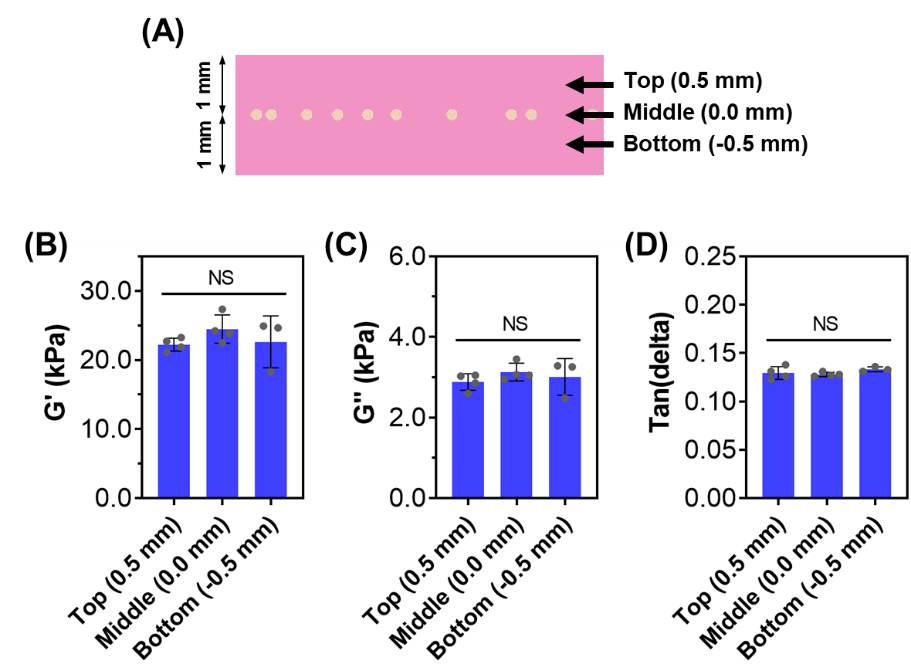


**Figure S3.** (A) Schematic illustration showing the nanoindentation coordinates at a hydrogel cross-section. (B) Storage moduli (G'), loss moduli (G''), and damping factor (tan(delta)) of FR hydrogels at different regions of the two-step casted samples. NS indicates no statistically significant difference (P > 0.05).


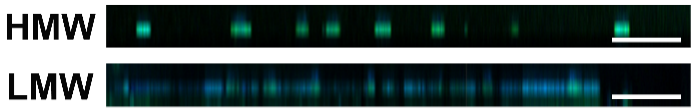


**Figure S4.** Representative cross-sectional views (y-z) of confocal z-stacks showing spheroids positioned within a plane after 5 days of culture within hydrogels made of high molecular weight (HMW) or low molecular weight (HMW) alginate. Scale bars = 500 µm.

**
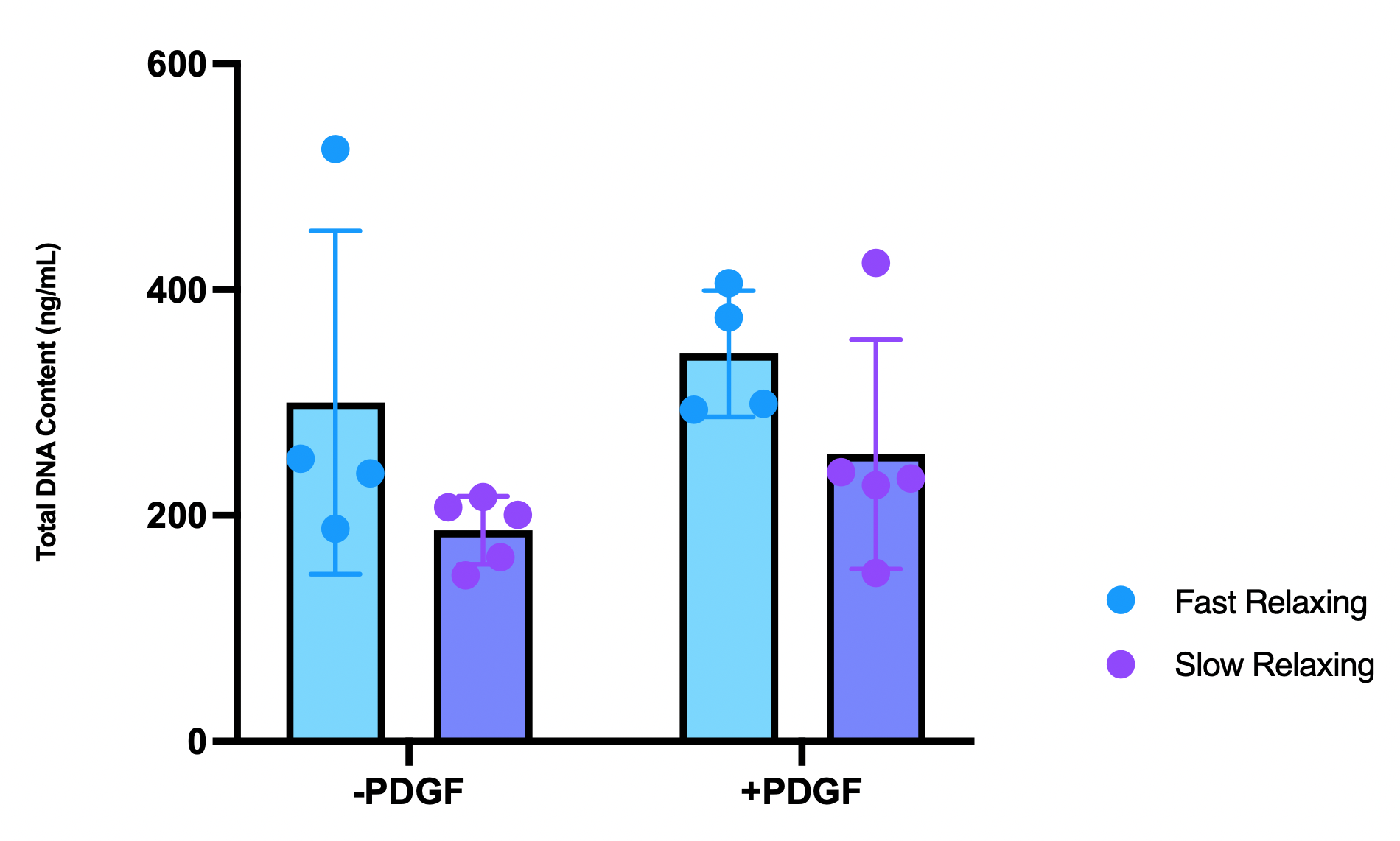
**

**Figure S5.** Total DNA content in spheroids cultured under different conditions for 5 days. Total DNA content (ng/mL) was used as an indirect marker of the extent of cell proliferation. No statistically significant differences were detected between conditions; Two-way ANOVA followed by Tukey's multiple comparisons test was applied.


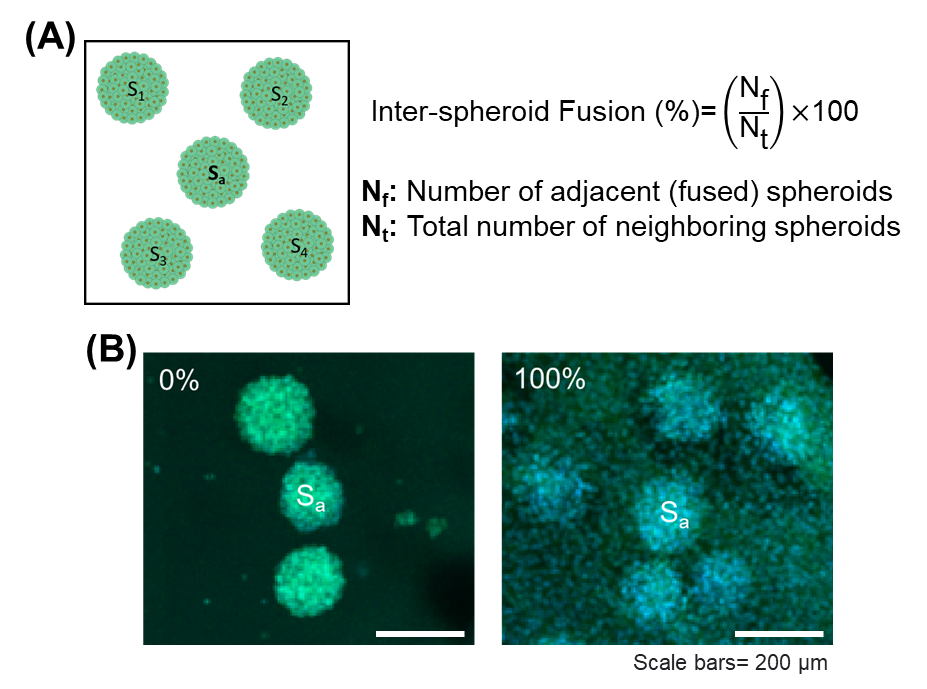


**Figure S6.** Overview of (A) the methodology used for quantification of inter-spheroid fusion and (B) examples of fused and non-fused spheroids.


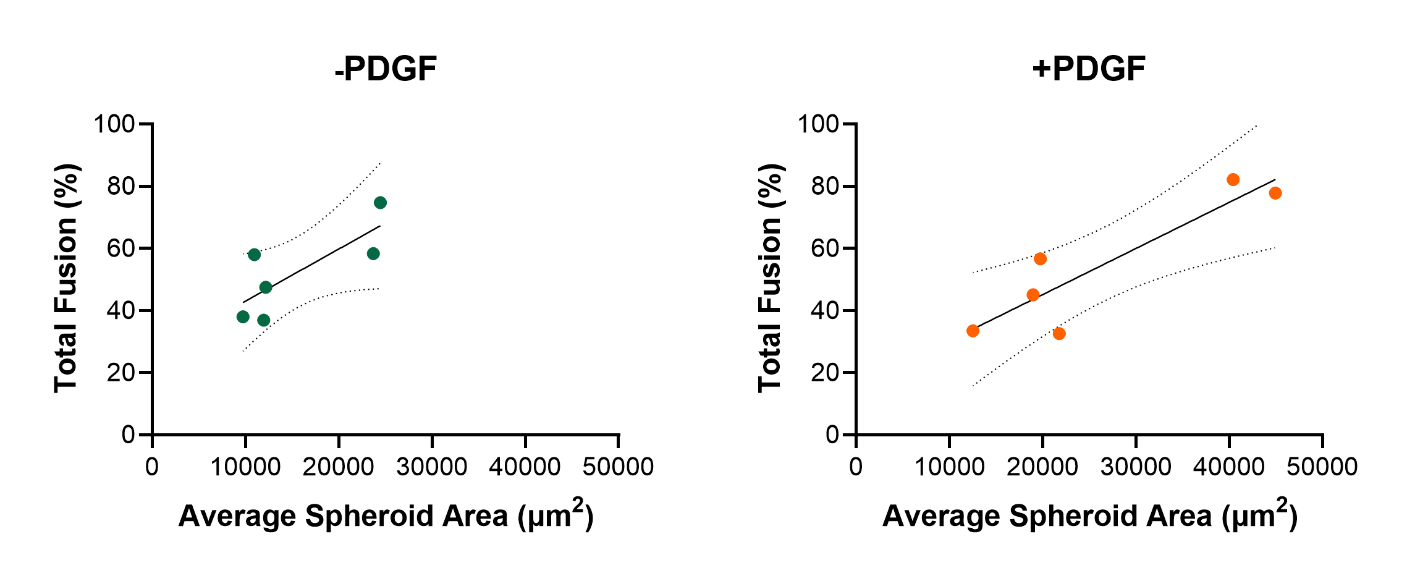


**Figure S7.** Total degree of fusion as function of average spheroid area for experimental groups cultured without (-PDGF) or with (+PDGF) supplementation. Each datapoint represents the mean value for an experimental group (compiled from 3-4 samples). Black continuous lines indicate the best-fit line of simple linear regression. Outer dashed lines indicate 95% confidence bands.
